# Correlations among peripheral blood markers, white matter hyperintensity, and cognitive function in patients with non-disabling ischemic cerebrovascular events

**DOI:** 10.3389/fnagi.2022.1023195

**Published:** 2022-12-02

**Authors:** Binghan Li, Bingying Du, Zhengsheng Gu, Chenghao Wu, Yuhao Tan, Chenrui Song, Yawen Xu, Ge Yin, Xin Gao, Weisen Wang, Xu Sun, Xiaoying Bi

**Affiliations:** Department of Neurology, Shanghai Changhai Hospital, The Second Military Medical University, Shanghai, China

**Keywords:** non-disabling ischemic cerebrovascular events, white matter hyperintensity, cognition, serum biomarker, vascular cognitive impairment

## Abstract

**Background:**

Both inflammation and cerebral white matter injury are closely associated with vascular cognitive impairment (VCI). The aim of this study was to analyze the correlation between peripheral serological markers, white matter injury, and cognitive function in patients with non-disabling ischemic cerebrovascular events (NICE); to identify potential biological markers for the diagnosis and prediction of VCI; and to provide a basis for the early diagnosis and intervention of VCI.

**Methods:**

We collected clinical data, along with demographic and medical history data, from 151 NICE patients. Fasting venous blood samples were collected. Based on the Montreal Cognitive Assessment (MoCA) after admission, we divided the patients into normal cognitive function (NCF) and VCI groups, and then classified them into mild white matter hyperintensity (mWMH) and severe white matter hyperintensity (sWMH) based on Fazekas scores. The differences in serological marker levels were compared between the cognitive function groups and the white matter hyperintensity groups. Binary logistic regression models and receiver operating characteristic curves were used to analyze the diagnostic predictive value of serological markers for VCI in patients with NICE and in the white matter hyperintensity subgroups.

**Results:**

Among 151 patients with NICE, 95 were male and 56 were female. Lymphocyte count (OR = 0.405, *p* = 0.010, 95% CI [0.201, 0.806]), red blood cell count (OR = 0.433, p = 0.010, 95% CI [0.228, 0.821]), and hemoglobin level (OR = 0.979, *p* = 0.046, 95% CI [0.958, 0.999]) were protective factors for cognitive function in patients with NICE. The sWMH group had a higher age, granulocyte/lymphoid ratio (NLR), and neutrophil percentage but a lower MoCA score, hemoglobin level, and lymphocyte count than the mWMH group. In the mWMH group, lymphocyte count (AUC = 0.713, *p* = 0.003, 95% CI [0.593, 0.833]) had an acceptable predictive value for the diagnosis of VCI, whereas white blood cell count (AUC = 0.672, *p* = 0.011, 95% CI [0.545, 0.799]), red blood cell count (AUC = 0.665, *p* = 0.014, 95% CI [0.545, 0.784]), and hemoglobin level (AUC = 0.634, *p* = 0.047, 95% CI [0.502, 0.765]) had marginal predictive value for the diagnosis of VCI. In the sWMH group, no significant differences were found in serological markers between the NCF and VCI groups.

**Conclusion:**

Lymphocyte count, red blood cell count, and hemoglobin level were independent protective factors for cognitive function in patients with NICE; they can be used as potential biological markers to distinguish VCI in patients with NICE and are applicable to subgroups of patients with mWMH.

## Introduction

Vascular cognitive impairment (VCI), the second most common form of cognitive impairment following Alzheimer’s disease, refers to cognitive impairment caused by cerebrovascular factors (van der Flier et al., 2018; Iadecola et al., 2019). The occurrence of VCI is closely related to cerebrovascular diseases (CVD; Zlokovic et al., 2020; Zanon Zotin et al., 2021), and several recent studies have demonstrated that brain ischemia, hypoxia, and endothelial damage due to CVD can lead to persistent inflammatory responses and white matter injury (Sigfridsson et al., 2020; Poh et al., 2022). In neuroimaging, white matter hyperintensities (WMHs) are one of the characteristic manifestations of white matter injury, and the severity of WMHs is positively correlated with the degree of cognitive decline in patients with CVD (Levit et al., 2020; Ungvari et al., 2021). In our previous study, we observed that the neutrophil-to-lymphocyte ratio (NLR) in the peripheral blood can reflect the level of central nervous system (CNS) inflammation to a certain extent (Liang et al., 2020). Furthermore, our findings indicated that the NLR was correlated with both mood and cognition in older female patients. Additional studies have indicated that the levels of serological inflammatory markers are closely related to the occurrence and prognosis of white matter injury and VCI; however, further studies are required to identify the inflammatory markers with the greatest predictive value for the diagnosis of VCI (Rosenberg, 2017; Simonetto et al., 2019).

Leukocytes, lymphocytes, and neutrophils are the most common peripheral blood markers of systemic inflammation (Guldolf et al., 2021; Ratter-Rieck et al., 2021; Buonacera et al., 2022). It has been suggested that these peripheral blood markers reflect systemic inflammation in patients and can serve as potential markers for the diagnosis of VCI (Lee et al., 2021). However, relevant studies have mainly focused on the correlation between inflammation and cognitive function, and no studies have analyzed the value of different degrees of white matter injury in the diagnosis and prediction of VCI (Zenaro et al., 2015; Magistrelli et al., 2020).

Non-disabling ischemic cerebrovascular events (NICE) represent the most common type of CVD in the Chinese population. To aid in identifying potential biomarkers for the diagnosis and prediction of VCI, the present study aimed to analyze the correlations among peripheral blood markers, white matter injury, and cognitive function in patients with NICE (Pan et al., 2019; Wang et al., 2021).

## Materials and methods

### Research participants

We consecutively enrolled and collected clinical data for 157 patients with NICE treated at Changhai Hospital (Shanghai, China) between January 2021 and February 2022. Among them, six patients who had not undergone head MRI were excluded; therefore, 151 patients were included in the final analysis. The inclusion criteria for patients with NICE were as follows: (a) National Institute of Health Stroke Scale Score (NIHSS) ≤3 at admission; (b) age 18–80 years; (c) first episode of NICE; (d) acute phase stroke with admission within 1 week of onset; and (e) no or only mild neurological deficits not exerting substantial effects on daily life or functioning.

The exclusion criteria were as follows: (a) inability to effectively complete the cognitive assessment (e.g., global aphasia, severe vision impairment, etc.); (b) history of organic mental disorder; (c) presence of cognitive impairments other than VCI; (d) history of inflammatory or autoimmune diseases; (e) history of malignant tumor; (f) severe liver or kidney dysfunction; and (g) refusal to participate in the study. This study was approved by the Ethics Committee of Changhai Hospital, and all participants provided written informed consent.

### WMH scoring

All patients underwent head MRI with T1-weighted (T1WI), T2-weighted (T2WI), and T2-weighted fluid-attenuated inversion recovery (T2WI-FLAIR) sequences. WMHs were grouped according to the distribution of hyperintensities in the paraventricular and deep white matter on T2WI using the Fazekas scale (0, normal; 1, mild; 2, moderate; 3, severe). Fazekas scores of 0 and 1 were defined as mild WMH (mWMH), while Fazekas scores of 2 and 3 were defined as severe WMH (sWMH). Imaging results were independently interpreted by two trained neurologists. Disagreements were resolved by a third senior physician.

### Assessment of neurocognition

Patients’ cognitive function was assessed using the Montreal Cognitive Assessment (MoCA) scale. All evaluators were trained in standardized scoring of the scale. VCI group was defined as MOCA scores less than 24. When the critical score is 24, MoCA has good specificity and sensitivity, which is supported by many studies based on Chinese population (Tsai et al., 2012; Chen et al., 2016; Liu et al., 2022); therefore, patients with a total score of <24 were diagnosed with cognitive impairment (Pugh et al., 2018). All patients included in this study were acute ischemic stroke patients, and for the patients from cognitive impairment group, all of them had a Hachinski Ischemic Scale greater than 7, which is consistent with the criteria of VCI. To further assess the cognitive domain, we used the following scales that have been shown to be valuable for the diagnosis of VCI, including the Auditory Verbal Learning Test (AVLT); Trail Making Test A/B (TMB-A/B); Symbol Digit Modalities Test (SDMT); Stroop Color-Word Test (SCWT); and Digit Span Test (DST) to assess working memory capacity (Skrobot et al., 2018).

### Statistical analysis

SPSS 24.0 (IBM, U.S.) was used for statistical analyses. Measurement data with normal distributions (Shapiro–Wilk Test) were expressed as mean ± standard deviation, and comparisons between groups were performed using independent samples *t*-tests or analyses of variance. Measurement data with a non-normal distribution were expressed as median (lower quartile, upper quartile), and comparisons between groups were performed using the Mann–Whitney U-test. Enumeration data were expressed as number and percentage, and comparisons between groups were performed using the *χ*^2^ test.

A binary logistic regression model was used to analyze the correlation between multiple biomarkers and cognitive function in patients with CVD. Moreover, after grouping based on WMH, patients with normal cognitive function (NCF) were used as the reference group to generate receiver operating characteristic (ROC) curves concerning the diagnostic value of relevant serological markers for VCI.

## Results

### Grouping

A total of 151 patients with NICE, including 95 men and 56 women, were included. The NCF and VCI groups included 79 and 72 patients, respectively. Based on Fazekas scores, the sWMH and mWMH groups included 70 and 81 patients, respectively.

### Comparisons between the VCI and NCF groups

Compared with the NCF group, the VCI group had a greater proportion of patients with sWMH (*p* < 0.05). In terms of serological markers, the VCI group had lower red blood cell count (*p* < 0.01, Cohen’s *d* = 0.43), hemoglobin level (*p* < 0.05, Cohen’s *d* = 0.35), and lymphocyte count (*p* < 0.01, Cohen’s *d* = 0.51). There were no significant differences in age, body mass index (BMI), sex, history of hypertension, diabetes, smoking, alcohol consumption, or other serological markers between the two groups (Table 1).

**Table 1:** Comparison of variables between the VCI and NCF groups.

Characteristics	VCI (*n* = 79)	NCF (*n* = 72)	*p*-value	Cohen’s *d*
Age (years)	66.00 (61.00, 70.75)	64 (56.75, 70.25)	0.115	0.319
BMI (kg/m^2^)	24.43 ± 3.23	24.72 ± 3.41	0.594	0.113
Gender, male, *n* (%)	46 (58.2%)	49 (68.1%)	0.212	
WMH, Severe, *n* (%)	50 (63.2%)	20 (27.8%)	**<0.001**	
Hypertension, *n* (%)	54 (68.4%)	52 (72.2%)	0.604	
Diabetes, *n* (%)	21 (26.6%)	22 (30.5%)	0.589	
Smoking, *n* (%)	35 (44.3%)	32 (44.4%)	0.904	
Alcoholism, *n* (%)	26 (32.9%)	21 (29.2%)	0.339	
Lymphocyte (*10^9^/L)	1.71 ± 0.49	1.95 ± 0.53	**0.008**	**0.510**
Lymphocyte percentage, %	28.93 ± 8.19	29.65 ± 7.70	0.585	0.118
Neutrophil (*10^9^/L)	3.73 (3.00,4.33)	3.80 (3.11,4.53)	0.526	0.095
Neutrophil percentage, %	60.69 ± 8.78	58.31 ± 8.30	0.093	0.306
NLR	2.15 (1.58,2.88)	2.00 (1.55,2.54)	0.168	0.175
Erythrocyte (*10^12^/L)	4.45 ± 0.54	4.69 ± 0.55	**0.010**	**0.430**
Hemoglobin (g/L)	136.16 ± 17.29	141.32 ± 14.23	**0.049**	**0.351**
WBC (*10^9^/L)	6.12 (5.05,7.22)	6.74 (5.30,7.61)	0.138	0.222
PLT (*10^9^/L)	204.00 (185.0,236.0)	205.00 (170.75,242.50)	0.675	0.015
AST(U/L)	18.00 (15.00,23.00)	19.00 (16.50,24.00)	0.222	0.234
ALT(U/L)	20.00 (15.00,26.75)	21.00 (16.00,29.00)	0.196	0.154
Uric acid (μmol/L)	334.26 ± 82.18	353.21 ± 99.99	0.205	0.202
Creatinine (μmol/L)	71.50 (61.00,82.50)	72.00 (60.50,83.00)	0.627	0.097
GFR (mL/min)	95.82 ± 22.47	95.60 ± 24.07	0.955	0.013
TG (mmol/L)	1.21 (0.95,1.73)	1.36 (0.97,2.20)	0.338	0.115
TC (mmol/L)	4.24 (3.61,5.11)	4.46 (3.96,5.25)	0.157	0.128
HDL-C (mmol/L)	1.22 (1.07,1.54)	1.22 (1.07,1.42)	0.492	0.165
LDL-C (mmol/L)	2.48 ± 0.83	2.69 ± 0.86	0.136	0.292
Fasting glucose (mmol/L)	5.25 (4.78,5.88)	5.30 (1.80)	0.984	0.058
Transferrin saturation, %	32.78 ± 12.60	32.94 ± 9.90	0.939	0.061
Serum iron (μmol/L)	16.78 ± 6.99	17.17 ± 4.44	0.724	0.112

WMH, White matter hyperintensities; BMI, body mass index; NLR, neutrophil-to-lymphocyte ratio; WBC, white blood cells; PLT, platelet; AST, aspartate aminotransferase; ALT, alanine transaminase; GFR, glomerular filtration rate; TG, triglyceride; TC, total cholesterol; HDL-C, high-density lipoprotein cholesterol; LDL-C, low-density lipoprotein cholesterol; MoCA, Montreal Cognitive Assessment; AVLT, Auditory Verbal Learning Test; SDMT, Symbol Digit Modalities Test; SCWT, Stroop Color-Word Test; DST, Digit Span Test.The bold value means *p*<0.05.

Logistic regression analysis adjusted for age and sex indicated that lymphocyte count (OR = 0.405, *p* = 0.010), red blood cell count (OR = 0.433, *p* = 0.010), and hemoglobin level (OR = 0.979, *p* = 0.046) were protective factors for cognitive function in patients with NICE. Meanwhile, WMHs were identified as a risk factor for cognitive decline in patients with NICE (Table 2).

**Table 2:** Variate prediction of different cognitive groups with binary logistic regression.

Variables		OR (95%CI)	*p*-value	Adjusted^a^ OR (95%CI)	*p* - value
Lymphocyte (*10^9^/L)		0.404 (0.202,0.806)	**0.010**	0.405 (0.201,0.806)	**0.010**
Erythrocyte (*10^12^/L)		0.443 (0.234,0.836)	**0.012**	0.433 (0.228,0.821)	**0.010**
Hemoglobin (g/L)		0.979 (0.959,0.999)	0.050	0.979 (0.958,0.999)	**0.046**
WMH		4.483 (2.250,8.932)	**<0.001**	4.463 (2.322,9.282)	**<0.001**

WMH, White matter hyperintensities.

a Adjusting for age and gender confounders.The bold value means *p*<0.05.

### Comparisons between the mWMH and sWMH groups

Patients in the sWMH group were significantly older than those in the mWMH group (*p* < 0.001). In addition, scores on the MoCA, AVLT, SDMT, SCWT, and DST (in reverse order) were lower in the sWMH group than in the mWMH group (all *p* < 0.001). In terms of serological markers, the sWMH group had lower hemoglobin level, lymphocyte count, serum iron level, transferrin saturation, and glomerular filtration rate (all *p* < 0.05). Meanwhile, NLR, fasting blood glucose level, and neutrophil percentage were significantly higher in the mWMH group than in the mWMH group (all *p* < 0.05). There were no significant differences in sex, BMI, history of hypertension, diabetes, smoking, or alcohol consumption or in other serological markers between the two groups (Table 3).

**Table 3:** Comparison of variables between the sWMH and mWMH groups.

Characteristics	sWMH (*n* = 70)	mWMH (*n* = 81)	*p*-value	Cohen’s *d*
Age (years)	68.00 (63.00,72.00)	63.00 (55.75,68.25)	**< 0.001**	**0.066**
BMI (kg/m^2^)	24.36 ± 3.17	24.75 ± 3.43	0.472	0.119
Gender, male, *n* (%)	42 (60.0%)	53 (65.4%)	0.491	
Hypertension, *n* (%)	50 (71.4%)	56 (69.1%)	0.759	
Diabetes, *n* (%)	23 (32.9%)	20 (24.7%)	0.268	
Smoking, *n* (%)	30 (42.8%)	37 (45.7%)	0.631	
Alcoholism, *n* (%)	21 (30.0%)	26 (32.1%)	0.818	
Lymphocyte (*10^9^/L)	1.76 ± 0.51	1.90 ± 0.52	0.117	0.277
Lymphocyte percentage, %	27.21 ± 6.98	31.01 ± 8.31	**0.003**	**0.501**
Neutrophil (*10^9^/L)	3.90 (3.40,4.38)	3.52 (2.70,4.51)	0.066	0.225
Neutrophil percentage, %	62.12 ± 7.96	57.33 ± 8.56	**0.001**	**0.587**
NLR	2.35 (1.86,3.06)	1.93 (1.52,2.39)	**0.002**	**0.361**
Erythrocyte (*10^12^/L)	4.47 ± 0.55	4.64 ± 0.55	0.063	0.312
Hemoglobin (g/L)	135.68 ± 15.87	141.15 ± 15.85	**0.037**	**0.350**
WBC (*10^9^/L)	6.42 (5.39,7.34)	6.34 (4.87,7.28)	0.361	0.155
PLT (*10^9^/L)	203.0 (181.0,241.0)	207.0 (172.0,237.0)	0.707	0.084
AST(U/L)	18.00 (16.00,24.00)	18.50 (14.75,22.25)	0.684	0.098
ALT(U/L)	21.00 (15.00,27.00)	20.00 (15.00,29.00)	0.983	0.086
Uric acid (μmol/L)	339.54 ± 92.66	346.60 ± 90.69	0.638	0.078
Creatinine (μmol/L)	72.00 (62.00,85.00)	71.5 (57.75,82.00)	0.286	0.212
GFR (mL/min)	91.37 ± 19.74	99.50 ± 25.30	**0.036**	**0.360**
TG (mmol/L)	1.22 (0.94,2.03)	1.30 (1.00,1.79)	0.694	0.025
TC (mmol/L)	4.42 (3.79,5.26)	4.37 (3.64,5.13)	0.582	0.134
HDL-C (mmol/L)	1.27 (1.08,1.55)	1.19 (1.05,1.40)	0.195	0.147
LDL-C (mmol/L)	2.58 ± 0.89	2.59 ± 0.81	0.971	0.008
Fasting glucose (mmol/L)	5.40 (4.83,6.38)	5.10 (4.70,5.80)	**0.029**	**0.154**
Transferrin saturation, %	30.28 ± 12.35	34.74 ± 10.22	**0.040**	**0.407**
Serum iron (μmol/L)	15.44 ± 6.80	18.08 ± 4.89	**0.026**	**0.466**
MoCA, score	21.00 (17.00,24.00)	25.00 (22.00,26.25)	**< 0.001**	**0.863**
AVLT, score	12.30 ± 5.79	16.68 ± 6.07	**< 0.001**	**0.750**
SDMT, score	23.73 ± 10.88	32.90 ± 12.07	**< 0.001**	**0.804**
SCWT, score	108.14 ± 42.14	85.8 ± 31.96	**< 0.001**	**0.620**
DST (inverted order)	4.00 (3.00,7.00)	6.00 (4.00,8.00)	**< 0.001**	**0.051**

BMI, body mass index; NLR, neutrophil-to-lymphocyte ratio; WBC, white blood cells; PLT, platelet; AST, aspartate aminotransferase; ALT, alanine transaminase; GFR, glomerular filtration rate; TG, triglyceride; TC, total cholesterol; HDL-C, high-density lipoprotein cholesterol; LDL-C, low-density lipoprotein cholesterol; MoCA, Montreal Cognitive Assessment; AVLT, Auditory Verbal Learning Test; SDMT, Symbol Digit Modalities Test; SCWT, Stroop Color-Word Test; DST, Digit Span Test.The bold value means *p*<0.05.

### Diagnostic and predictive value of serological markers for VCI in different WMH groups

Of the 81 patients with mWMH, 29 patients were in the VCI group. Red blood cell count (*p* = 0.006), hemoglobin level (*p* = 0.047), white blood cell count (*p* = 0.024), lymphocyte count (*p* = 0.002), and neutrophil count (*p* = 0.033) were lower in the VCI group than in the NCF group. The logistic regression analysis of the above indicators revealed that, after adjusting for sex and age, lymphocyte (OR = 0.241, *p* = 0.014) and red blood cell (OR = 0.291, *p* = 0.039) counts were protective factors for cognitive function in patients with NICE (Table 4).

**Table 4:** Variate prediction of different cognitive groups in mWMH with binary logistic regression.

Variables	OR (95%CI)	*p*-value	Adjusted^a^ OR (95%CI)	*p*-value
Lymphocyte(* 10^9^/L)	0.241 (0.077,0.752)	**0.014**	0.241 (0.077,0.752)	**0.014**
Erythrocyte(* 10^12^/L)	0.291 (0.090,0.939)	**0.039**	0.291 (0.090,0.939)	**0.039**

WMH, White matter hyperintensities.

a Adjusting for age and gender confounders.The bold value means *p*<0.05.

**Figure 1:**
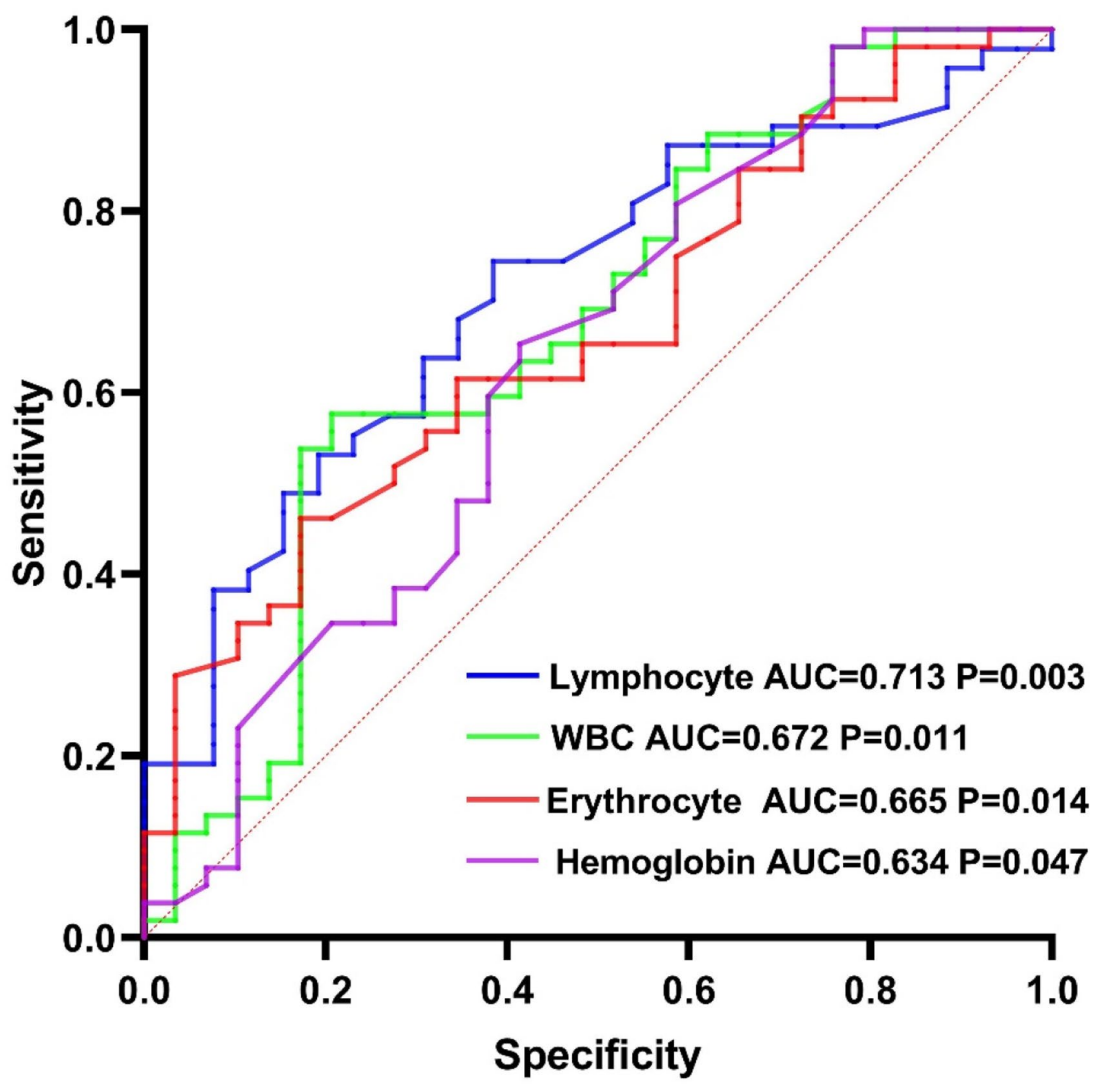
Diagnostic and predictive value of serological markers for vascular cognitive impairment in the mild white matter hyperintensity group.

We also generated ROC curves to explore the indicators with predictive and diagnostic value for VCI in patients with mWMH. Among them, lymphocytes had the highest predictive value (AUC = 0.713, *p* = 0.003, 95% CI [0.593, 0.833], cutoff point: 1.73 × 10^9^/L, sensitivity: 74.47%, specificity: 61.54%; Figure 1).

Among patients with sWMH, the VCI group had fewer years of education, although there were no significant differences in any serological markers between the VCI and NCF groups (*p* > 0.05).

## Discussion

In recent years, the incidence of cognitive impairment in China has gradually increased, with VCI accounting for 20–40% of all types of cognitive impairment (van der Flier et al., 2018). VCI is known to exert a serious impact on both quality of life and mortality in affected patients (Rundek et al., 2022), highlighting the importance of identifying diagnostic indicators that can aid in early clinical intervention. In this study, we investigated the associations among serological markers, WMH, and VCI by examining serological marker levels in different WMH groups and their associations with different levels of cognitive function in patients with NICE.

Our findings indicated that patients with VCI had lower lymphocyte count, and lymphocyte counts were an independent protective factor for cognitive function in patients with CVD. Studies have shown that peripheral inflammation can cause damage to the blood–brain barrier and that peripheral immune cells can infiltrate into the brain parenchyma, leading to neuroinflammation (Bowman et al., 2018; Shen et al., 2019). In the process of neuroinflammation, the continuous production of inflammatory factors can lead to various types of damage to the nervous system, including endothelial dysfunction, vascular aging, blood–brain barrier disruption, amyloidosis, neuronal death, and CVD—which all contribute to impairments in cognitive function (Simonetto et al., 2019; Rajeev et al., 2022). When the peripheral immune system is activated, immune cells transform into a proinflammatory phenotype, following which they release proinflammatory factors into the brain and peripheral blood, which can in turn influence cognitive function (Mietelska-Porowska and Wojda, 2017; Aruldass et al., 2021). In accordance with our results, previous studies have reported that lymphocyte counts are predictors of cognitive function (Kim et al., 2012; Ren et al., 2017). This may be due to the apoptosis of lymphocytes during cerebrovascular events. Such events result in transformation from the TH-1 type, which promotes inflammatory responses, to the anti-inflammatory TH-2 type, *via* stimulation by growth factor-18 after demyelination (Shim and Wong, 2016). Meanwhile, lymphocytes are known to regulate neuroinflammation following cerebrovascular events and promote white matter repair by secreting interleukin-10, a key anti-inflammatory and neuroprotective cytokine, thus leading to cognitive recovery (Tokgoz et al., 2014; Liesz et al., 2015; Nam et al., 2017). The results of this study indicate that lymphocyte-mediated inflammatory responses have important clinical value for predicting and possibly preventing cognitive decline in patients with NICE.

Our results also indicated that patients with VCI had lower red blood cell counts and hemoglobin levels than those without and that red blood cells and hemoglobin were independent protective factors for cognitive function in patients with NICE, consistent with the findings of Lauriola et al. (2018). Hemoglobin carries oxygen to meet the basic nutritional needs of blood vessels and nerves, and anemia is a known risk factor for cognitive impairment, although the exact mechanism remains unclear (Wolters et al., 2019). Research has demonstrated that red blood cells contain many important bioactive mediators, which play an important role in the CNS. However, under the influence of vascular risk factors or cerebrovascular events, the balance among these red blood cell components is disturbed, leading to changes in membrane surface receptors, which are closely related to the occurrence and development of VCI (Hassouna et al., 2016; Graham et al., 2019; Sitzia et al., 2020; Wakhloo et al., 2020). The current results indicate that hemoglobin-and red blood cell-related functions have important value and significance for cognitive decline in patients with NICE.

The present results also demonstrated that scores on the MoCA, AVLT, SDMT, SCWT, and DST (in reverse order) were lower in the sWMH group than in the mWMH group. This finding suggests that patients with white matter injury exhibit impairments in executive function, memory, attention, and response inhibition, which have been demonstrated in several previous studies (Kynast et al., 2018; Pantoni et al., 2019). However, the mechanisms by which WMHs lead to cognitive decline remain unclear. Inflammation, endothelial dysfunction, changes in blood–brain barrier permeability, and myelin degeneration may play key roles in this process (Wardlaw et al., 2019; Hu et al., 2021). Previous work by our group has shown that oligodendrocyte precursor cells, which are the basis of normal myelination, are vulnerable to damage under mild ischemia and hypoxia, following which they release inflammatory factors with impaired differentiation and maturation profiles, thereby resulting in white matter injury and affecting cognitive function (Bi et al., 2012; Du et al., 2020). In this study, we observed that patients with sWMH had higher neutrophil counts and NLR and lower lymphocyte percentages than those with mWMH. This result suggests that neutrophil count, NLR, and lymphocyte count are potential serological markers of white matter injury.

The results of this study indicate that lymphocytes and red blood cells are protective factors for cognitive function in patients with NICE who have mWMH. At the same time, ROC curves based on the presence or absence of VCI indicated that lymphocytes, red blood cells, white blood cells, and hemoglobin had good value in the prediction and diagnosis of VCI in patients with mWMH, with lymphocyte count representing the most predictive factor. However, no serological markers exhibited significant role in cognitive function in patients with sWMH. According to a study by Kynast, which compared patients with severe white matter injury to those with mild white matter injury, most neural structures remained intact in cases of mild injury. Although the efficiency of communication within the nervous system is reduced, neurotransmission and metabolic functions may be preserved in such cases (Kynast et al., 2018). Moreover, in most studies, calcium overloading, oxidative stress, blood–brain barrier dysfunction and neuroinflammation were found to be important mediators between leukoencephalopathy and VCI. In patients with mild white matter injury, the related mechanisms mediated by neuroinflammation play a major role (such as signal transduction by the Receptor for Advanced Glycation End Products (RAGE)). But in patients with severe white matter injury, the underlying mechanisms leading to cognitive dysfunction are more complex. Other mechanisms, not just neuroinflammation, may play a major role in the development of VCI such as: 1. Neurotoxic molecules pass through the damaged blood–brain barrier and lead to neuronal damage; 2. Elevation in reactive oxygen species (ROS) along with decreased nitric oxide (NO) and antioxidant enzymes lead to mitochondrial metabolic disorders, which predisposes neuronal dysfunction and apoptosis (Hase et al., 2018; Sweeney et al., 2019; Chen et al., 2021; Yang and Zhang, 2021). This may also be the reason why we did not find cognition-related differential inflammatory markers in patients with sWMH, and that’s why we call for early screening with neuroinflammatory markers. A study by Tao et al. indicated that red blood cells were protective factors for VCI in patients with severe white matter injury, although the authors reported no protective effect in patients with mild white matter injury. This may be due to immune adhesion and phagocytosis mediated by complements and receptors on the surface of red blood cells, which are closely related to cognitive function, beginning to take effect as the severity of white matter injury increases (Tao et al., 2022). These results also indicate that the specific relationship between the degree of white matter injury and that of cognitive decline remains to be investigated (Vignini et al., 2019). Based on our results, lymphocyte count, red blood cell count, white blood cell count, and hemoglobin level exhibited significant values for predicting and evaluating VCI in patients with mild white matter injury, with lymphocyte count having the highest value.

The present study has some limitations, including its cross-sectional design, which cannot be used to reveal causal relationships. Thus, longitudinal studies are needed to confirm our findings. In addition, all serological markers were tested only once, indicating that laboratory measurement errors may have affected the accuracy of the data. Lastly, the sample size was small, and the results may have been biased.

In conclusion, our findings indicated that lymphocyte count, red blood cell count, and hemoglobin level were independent protective factors for cognitive function in patients with NICE. Further, our analysis identified neutrophil count, NLR, and lymphocyte count as potential serological markers of white matter injury. Lymphocytes and red blood cells were specifically observed as protective factors for cognitive function in patients with NICE exhibiting mWMH. Further, lymphocyte count, red blood cell count, white blood cell count, and hemoglobin level exhibited good predictive values for the diagnosis of VCI in patients with mWMH, with lymphocyte count exhibiting the highest value. However, no valuable serological markers were identified in the sWMH group. In the future, prospective studies with larger sample sizes are needed to further explore the associations among VCI, serologic inflammatory markers, and WMH.

## Data availability statement

The raw data supporting the conclusions of this article will be made available by the authors, without undue reservation.

## Ethics statement

This study was approved by the Ethics Committee of Changhai Hospital, and all participants provided written informed consent.

## Author contributions

BL, XS, and XB conceived and designed the study. BL, BD, ZG, YT, CW, CS, YX, XS, and XB performed the study. BL, BD, WW, CW, GY, XG, and XS revised the article for intellectual content. BD and BL wrote the article. All authors contributed to the article and approved the submitted version.

## Funding

This work was supported by grants from the Shanghai Health System Talent Training Program (2018BR29), Major clinical research projects of Shanghai ShenKang Hospital Development Center (SHDC2020CR1038B), and Scientific research project of Shanghai Health Commission (20214Y0500).

## Conflict of interest

The authors declare that the research was conducted in the absence of any commercial or financial relationships that could be construed as a potential conflict of interest.

## Publisher’s note

All claims expressed in this article are solely those of the authors and do not necessarily represent those of their affiliated organizations, or those of the publisher, the editors and the reviewers. Any product that may be evaluated in this article, or claim that may be made by its manufacturer, is not guaranteed or endorsed by the publisher.
